# Effect of cardiac rehabilitation therapy on depressed patients with cardiac insufficiency after cardiac surgery

**DOI:** 10.1515/med-2023-0821

**Published:** 2023-11-14

**Authors:** Wenyu Zhang, Guangjian Zhu, Beibei Li, Chen Chen, Yingzhi Zhu

**Affiliations:** Department of Cardiology, Affiliated Nanjing Brain Hospital, Nanjing Medical University, Nanjing, Jiangsu, 210029, China

**Keywords:** cardiac rehabilitation therapy, cardiac surgery, anxiety and depression, cardiac autonomic function, cardiac insufficiency

## Abstract

This study aims to analyze the effect of cardiac rehabilitation therapy on cardiac autonomic nervous function in patients with cardiac insufficiency complicated with anxiety depression after cardiac operation to provide a reference for clinical practice. A total of 109 patients subject to cardiac operation in our hospital from January 2020 to March 2023 were enrolled as study subjects, including 50 patients who received conventional rehabilitation therapy (control group) and 69 patients who received cardiac rehabilitation therapy (research group). Before and after treatment, the left ventricular ejection fraction (LVEF) and central venous pressure (CVP) were determined, and the level of N-terminal pro-B-type natriuretic peptide (NT-proBNP) was measured. Low frequency/high frequency (LF/HF), standard deviation of normal to normal (SDNN), and root mean of successive square differences (RMSSD) were measured by a multi-lead ECG system. Self-rating Anxiety Scale (SAS), Self-rating Depression Scale (SDS), Pittsburgh Sleep Quality Index (PSQI), Activity of daily living (ADL), and Barthel Index (BI) were applied for corresponding investigations, as well as the 6-min walk test (6MWT). After treatment, the research group showed higher LVEF, CVP, LF/HF, SDNN, and RMSSD, and lower NT-proBNP, SAS, and SDS than the control group (*P* < 0.05). Significantly elevated ADL score, BI, and 6MWT and reduced PSQI were observed in both groups after treatment, with more remarkable changes in the research group (*P* < 0.05). In conclusion, cardiac rehabilitation therapy effectively improved the cardiac function of patients with cardiac insufficiency complicated with anxiety and depression after the cardiac operation and alleviated their negative emotions.

## 1 Introduction

Cardiovascular disorder is one of the most common types of diseases in clinical practice, and its mortality rate is also among the highest in all diseases [1]. According to the World Health Organization (WHO) in 2015, out of the 56 million deaths worldwide, the number of deaths caused by cardiovascular disorders has reached 17.7 million, and the number of deaths due to cardiovascular disorders is estimated to increase by 6 million every year [2]. Cardiac operation is also increasingly used clinically as a treatment approach for many cardiovascular disorders [3]. After cardiac operation, cardiac insufficiency due to invasive operations is the most common pathological injury, which greatly affects the prognosis and recovery of patients, and increases the possibility of risk events such as arrhythmia and cardiac failure [4]. Patients’ negative emotions occurring after diseases and operations also need attention. According to statistics, more than 70% of the patients with a history of cardiac operation experience varying degrees of post-operative depression [5]. The occurrence of such negative emotions is detrimental to patient prognosis and recovery and may increase the cardiac preload and affect the pumping function of the heart [6]. Therefore, postoperative intervention is also important in managing the cardiac disorder of patients.

Psycho-cardiology is a sub-discipline of psychosomatic medicine and a sub-specialty of cardiology, which refers to the treatment of patients with both heart disorders and psychic disorders; it advocates not only paying attention to the heart but also to the psychology of patients, so as to achieve “mind-body” coordination and to jointly resolve the physical pain and psychological trauma of patients through multi-level interventions [7,8]. Although cardiac rehabilitation therapy has made many achievements in clinical practice [9,10], there are few reports on its value in improving the cardiac autonomic nervous function in patients with cardiac insufficiency complicated with anxiety and depression after cardiac operation. In order to further clarify its application effect, a detailed study is carried out, and the results are as follows.

## 2 Materials and methods

### 2.1 Sample size calculation

The sample size was calculated according to the sample size formula *N* = *Z*² × [*P* × (1–*P*)]/*E*², with the statistic (*Z*) set at 1.64, the probability value (*P*) set at 0.5, and the error value (*E*) set at 10%. The sample size (*N*) was obtained as 96, i.e., a minimum of 96 subjects were required for this study.

### 2.2 Clinical data of patients

Cardiac patients admitted to Affiliated Nanjing Brain Hospital from January 2020 to March 2023 were selected for the study, and after screening according to the inclusion criteria (patients clinically diagnosed with heart disorder by imaging and typical symptoms, with indications for interventional procedures; patients with postoperative cardiac insufficiency complicated with anxiety depression [all patients met the diagnostic criteria for depression in the International Classification of diseases, ICD-10, all patients in this study were mildly depressed]; patients informed of participation in this study; patients with high compliance; patients with complete clinical data; patients with good basic status), 241 patients were initially identified. After screening according to the exclusion criteria (patients with serious unstable or life-threatening conditions after operation; patients complicated with other cardiac disorders; patients with previous history of cardiac or thoracic operations; patients who used anti-anxiety, anti-depression or central nervous system sedative drugs; patients with poor compliance; patients with mental or consciousness disorders, or communication disorders), 119 patients were finally identified. The study population was cardiac patients who were referred to our cardiology department for cardiac rehabilitation within 3–7 days after surgery in other hospitals. The control group consisted of 50 patients who received conventional rehabilitation therapy after cardiac operation, and the research group consisted of the other 69 patients who received cardiac rehabilitation therapy after cardiac operation. All subjects were followed up by the same research team members (Table 1).

**Table 1:** Comparison of general inspection data

Group	Age	Gender		Marital status		Type of disease				
		Male	Female	Unmarried(or divorced)	Married	Coronary heart disease	Heart valve disease	Myocardial infarction	Heart failure	Other
Control group (*n* = 50)	70.36 ± 13.21	34 (68.00)	16 (32.00)	10 (20.00)	40 (80.00)	26 (52.00)	14 (28.00)	3 (6.00)	4 (8.00)	3 (6.00)
Research group (*n* = 69)	72.12 ± 9.86	47 (68.12)	22 (31.88)	8 (11.59)	61 (88.41)	40 (57.97)	16 (23.19)	3 (4.35)	7 (10.14)	3 (4.35)
*t* (or *χ* ^2^)	0.831	<0.001		1.596		0.911				
*P*	0.408	0.983		0.207		0.923				

### 2.3 Intervention schedule

Conventional rehabilitation therapy: The rehabilitation therapy was not performed until the patient’s condition was stable after the operation, within 24 h after the operation (or at the earliest time that the patient can tolerate). Once the patients were able to move independently, active exercises such as stretching the upper and lower limbs and deep breathing were to be completed on the bed. During the rehabilitation process, the patients’ heart rate and blood pressure were strictly monitored; the rehabilitation therapist assessed the patients’ exercise ability and recommended appropriate exercises for the patients according to his/her condition. Cardiac rehabilitation therapy: first, a cardiac rehabilitation therapy team was established. Team members were required to be familiar with the intervention schedule, understand the concept of cardiac rehabilitation, learn communication skills, and develop intervention measures according to the condition of each patient. Besides, the team members communicated with patients’ families about the completion of the operation, and encouraged the family members to accompany and encourage the patients; informed the patients about previous successful rehabilitation cases to enhance their self-confidence and improve rehabilitation compliance; promptly paid attention to the patients’ inner needs to provide corresponding assistance and support; followed the doctor’s advice to instruct the patients on postoperative medication; and informed the patients of the importance of postoperative rehabilitation training to help them establish a correct postoperative cognition. At the same time, staged rehabilitation exercises were carried out according to the physical conditions of each patient, from simple physical activities to short time exercises requiring endurance. During the process, the patients were monitored for heart rate and blood pressure, and the exercise plan was adjusted in time. Patients with positive attitudes or efforts to participate in rehabilitation exercises were complimented and taken as role models for other patients. Besides, the patients were instructed on negative emotion alleviation by meditation, music therapy, decompression via deep breathing, relaxation of whole body muscles, and other self-relief methods, and were encouraged to participate in group activities to divert attention and to establish a good state of mind to maintain a relaxed state. In addition, all patients need to be counseled by a psychiatrist: attention is paid to the psychological state of patients, and psychologists are regularly scheduled to provide psychological counseling treatment to patients weekly. Continuous intervention for 2 weeks was considered as a course of treatment, for a total of 2 courses of treatment.

### 2.4 Outcome measures

Before and at 4 weeks after treatment, the left ventricular ejection fraction (LVEF) and central venous pressure (CVP) were determined, and elbow venous blood was collected under fasting state to measure the level of N-terminal pro-B-type natriuretic peptide (NT-proBNP) in both groups. Low frequency/high frequency (LF/HF), standard deviation of normal to normal (SDNN), and root mean of successive square differences (RMSSD) were measured by a multi-lead ECG system (MECG-300, Medex, Beijing). Before measurement, patients were required to rest quietly for 15 min. Such measurement was performed by the same senior professionals for three times, and the results were averaged. Self-rating Anxiety Scale (SAS) and Self-rating Depression Scale (SDS) [11] were used to evaluate the patients’ anxiety and depression, and the scores were positively correlated with the degree of anxiety and depression. The Pittsburgh Sleep Quality Index (PSQI) [12] was applied to evaluate the sleep quality of patients, and the score was negatively correlated with the sleep quality. Activity of Daily Living (ADL) [13] and Barthel Index (BI) [14] were applied to evaluate the patients’ living ability, and the results were positively correlated with self-care ability. The 6-min walking test (6MWT) was applied to assess the patients’ mobility.

### 2.5 Statistical methods

Statistical analysis was performed using SPSS23.0 software. Measurement data were expressed as mean ± standard deviation ( \[\bar{\chi }]\] ± s) and compared using independent samples *t*-test and paired *t*-test. The Enumeration data were expressed as percentages (%) and compared using the chi-square test. *P* < 0.05 indicated statistically significant differences.


**Ethics approval:** The study was approved by the Ethics Committee of Affiliated Nanjing Brain Hospital (No.2014KY039).

## 3 Results

### 3.1 Comparison of cardiac function

**Figure 1:**
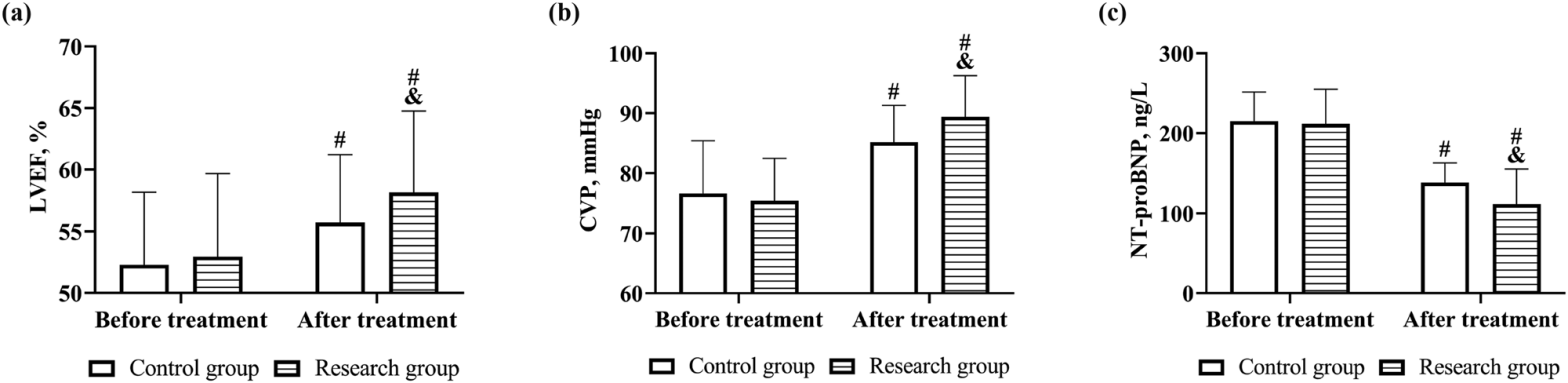
Cardiac function before and after treatment in both groups. Comparison of (a) LVEF, (b) CVP, and (c) NT-proBNP. Note: vs before treatment ^#^ *P* < 0.05, vs control group ^&^ *P* < 0.05.

Before treatment, there was no difference in LVEF, CVP, and NT-proBNP between the two groups (*P* > 0.05). After treatment, LVEF and CVP increased in both groups, and were higher in the research group; while NT-proBNP decreased, and was lower in the research group (*P* < 0.05, Figure 1).

### 3.2 Comparison of cardiac autonomic nervous function

**Figure 2:**
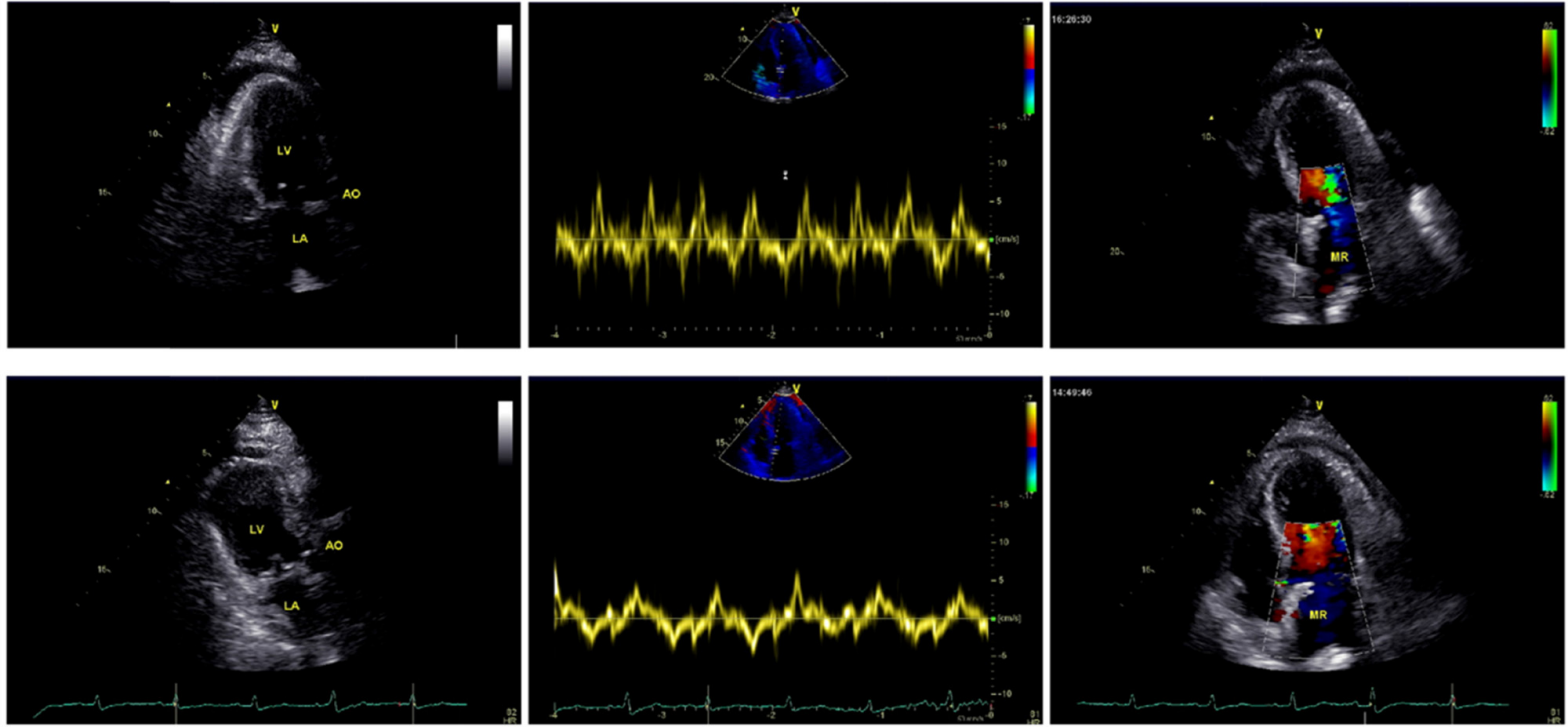
Comparison of cardiac ultrasound findings before and after treatment in a patient (male, 57 years old); the first row is before treatment and the second row is after treatment.

**Figure 3:**
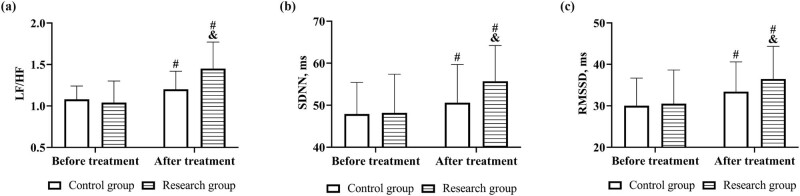
Cardiac autonomic nervous function before and after treatment in both groups. Comparison of (a) LF/HF, (b) SDNN, and (c) RMSSD. Note: vs before treatment ^#^ *P* < 0.05, vs control group ^&^ *P* < 0.05.

Similarly, there was no difference in the results of cardiac autonomic nervous function tests between the two groups before treatment (*P* > 0.05). LF/HF, SDNN, and RMSSD elevated in both groups after treatment and were higher in the research group (*P* < 0.05, Figures 2 and 3).

### 3.3 Comparison of psychological status

**Figure 4:**
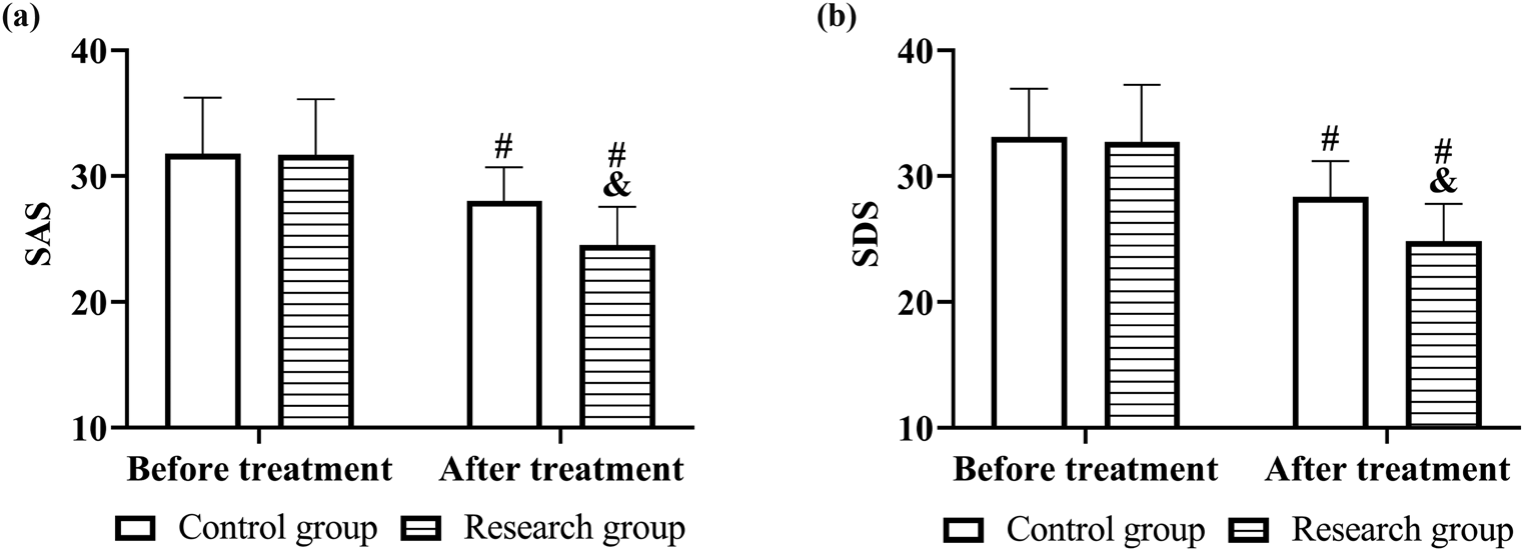
Psychological status before and after treatment in both groups. Comparison of (a) SAS and (b) SDS. Note: vs before treatment ^#^ *P* < 0.05, vs control group ^&^ *P* < 0.05.

After treatment, SAS and SDS decreased to 24.51 ± 3.02 and 24.80 ± 2.99 in the research group and 28.02 ± 2.66 and 24.80 ± 2.99 in the control group, respectively, and both of them were lower in the research group (*P* < 0.05, Figure 4).

### 3.4 Comparison of self-care ability

**Figure 5:**
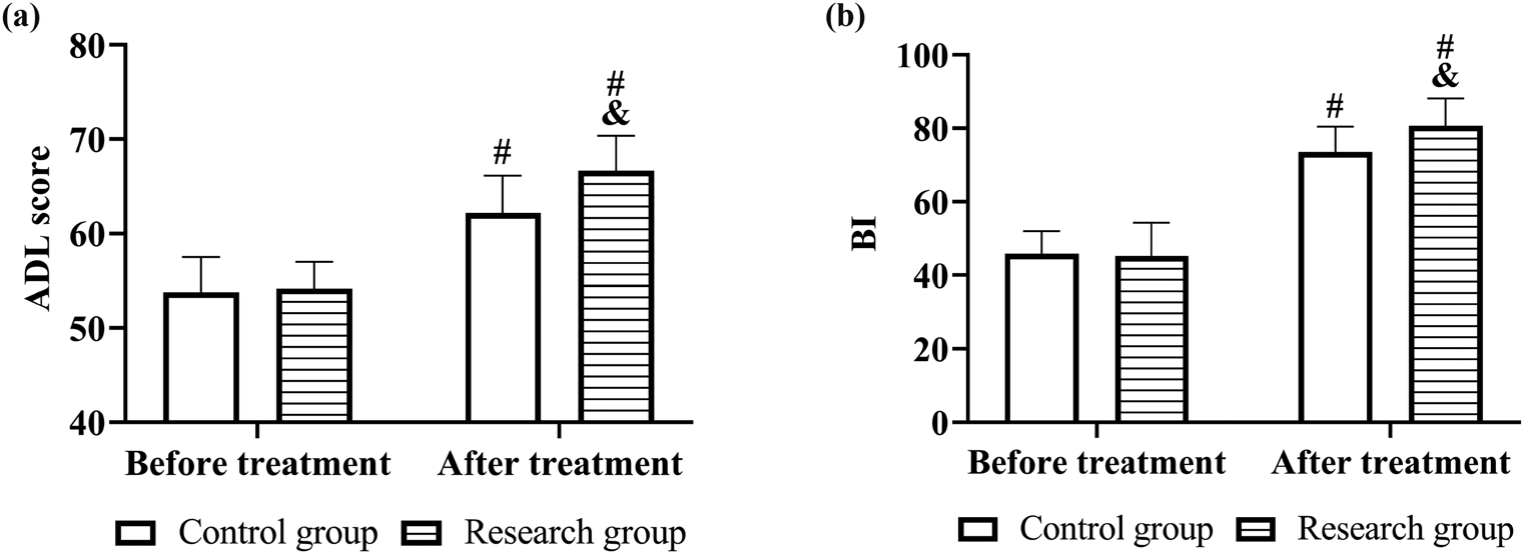
Self-care ability before and after treatment in both groups. Comparison of (a) ADL score and (b) BI. Note: vs before treatment ^#^ *P* < 0.05, vs control group ^&^ *P* < 0.05.

Before treatment, there was no statistically significant difference in ADL score and BI between the two groups (*P* > 0.05). After treatment, ADL score and BI increased significantly in both groups, with a more obvious trend of increase in the research group (*P* < 0.05, Figure 5).

### 3.5 Comparison of sleep quality and activity

**Figure 6:**
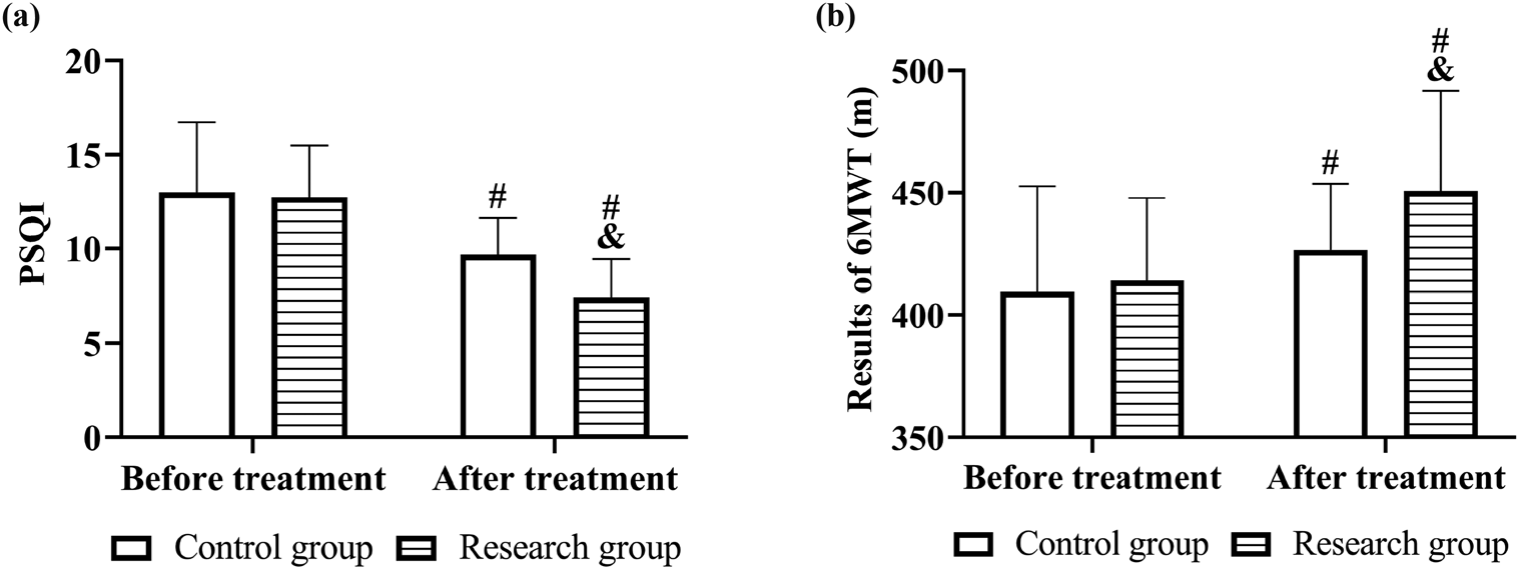
Sleep quality and activity before and after treatment in both groups. Comparison of (a) PSQI and (b) 6MWT results. Note: vs before treatment ^#^ *P* < 0.05, vs control group ^&^ *P* < 0.05.

The results of PSQI and 6MWT were compared between the two groups. Compared with the control group, the research group had a lower PSQI score and better 6MWT result (*P* < 0.05). Both groups showed decreased PSQI scores and better 6MWT results after treatment (*P* < 0.05, Figure 6).

## 4 Discussion

In this study, patients were first compared in terms of cardiac function, and both LVEF and CVP of patients in the research group were higher than those in the control group. As cardiac rehabilitation mode improves patients’ endurance for activities by organizing training such as breathing mindfulness and relaxation mindfulness, reduces the concentration of 5-hydroxytryptamine in patients, inhibits the secretion of inflammatory factors in the body, enhances the biological activity of nitric oxide synthase, decreases nitric oxide content and catecholamine secretion, reduces vascular endothelial damage, suppresses the development of atherosclerosis, improves vascular diastolic function and the windkessel effect, and reduces cardiac preload, and therefore, the cardiac pumping function is improved [15,16]. In addition, in previous studies, researchers also suggested that the cardiac rehabilitation mode improves physiological activity in the hippocampus and prefrontal cortex through the hypothalamic–pituitary–adrenal axis, inhibits the overactivated sympathetic nervous system while appropriately activating the parasympathetic nervous system, enhances autonomic nervous regulation function, and restores patients’ circadian rhythm, which has a positive effect on the regulation of neurological function [17]. When cardiac autonomic function was examined in both groups, a superior cardiac autonomic function was also observed in the research group, which verifies the point above.

In the investigation of the psychological status of patients in both groups, SAS and SDS scores in the research group after rehabilitation therapy decreased more remarkably than those in the control group, which also indicated that cardiac rehabilitation therapy effectively alleviated the negative emotions of patients, consistent with the findings of García-Bravo et al. [18]. Such effect was achieved by simultaneous psychological interventions and conventional physiological care in cardiac rehabilitation therapy so that patients achieved physical and psychological coordination. Weak status and inability to move in the early postoperative period lead to high psychological stress in patients, which gives rise to more severe anxiety, depression, and other negative emotions [19]. In cardiac rehabilitation therapy, the nursing staff explained to patients that postoperative weakness was a normal phenomenon through a friendly and patient attitude, and introduced the following rehabilitation schedule to the patients, so that the patients received and felt professional support, and the confidence in recovery was therefore improved. Since patients are in a special period, their family members are guided to communicate more with them and encourage them to express their inner demands, so that they can feel adequate family support, and their psychological pressure is relieved [20]. In addition, cardiac rehabilitation therapy also requires the nursing staff to introduce the dosage and time of administration of each drug to patients during drug distribution, and to explain the effects of each drug, thus enhancing patients’ medication adherence [21]. As mentioned above, cardiac rehabilitation therapy helps to improve the cardiac autonomic function of patients, which is also beneficial to regulate their dopamine secretion in the body; relieve their reflexive anxiety, panic, anger, and other adverse emotions; and finally relieve all of their negative emotions. Also, this view should be verified through more basic experiments.

A comparison of postoperative activity between the two groups also showed superior results in the research group, which further proves the positive role of cardiac rehabilitation therapy in cardiac operation. Cardiac rehabilitation therapy adopts different rehabilitation means based on different stages of patients’ postoperative recovery; when patients cannot get out of bed and move, passive training is applied to help them move their limbs, followed by bedside activities and aerobic training as they gradually get better so that their recovery is promoted by real-time rehabilitation schedule adjustment based on their recovery status [22,23]. Through the alleviation of patients’ negative emotions, cardiac rehabilitation therapy also allows patients to have reasonable expectations of their own recovery, so that they will have confidence in the rehabilitation process and participate in the rehabilitation work more actively, which ensures the effect of rehabilitation exercise and facilitates postoperative recovery.

## 5 Conclusion

Cardiac rehabilitation therapy effectively improved the cardiac function of patients with cardiac insufficiency complicated with anxiety and depression after cardiac operation, alleviated their negative emotions, fastened their recovery after operation, and improved the therapeutic effect and safety of operation. We believe that in the future, cardiac operation will be applied in the clinic in combination with cardiac rehabilitation therapy to provide patients with more scientific and effective treatment services.

Due to the limited experimental conditions, there are several limitations in this study. For example, the number of cases included in this study is small, making the results not comprehensive and representative enough. Moreover, since there are no unified clinical guidelines for cardiac rehabilitation therapy, there is still room for optimization and improvement in detail.

## Appendix

**Table A1:** Cardiac function before and after treatment in both groups

		Control group (*n* = 50)	Research group (*n* = 69)	*t*	*P*
LVEF (%)	Before treatment	52.30 ± 5.86	52.96 ± 6.72	0.558	0.578
	After treatment	55.72 ± 5.49^*^	58.14 ± 6.62^*^	2.111	0.037
CVP (mmHg)	Before treatment	76.62 ± 8.78	75.45 ± 7.04	0.806	0.422
	After treatment	85.14 ± 6.18^*^	89.41 ± 6.88^*^	3.489	<0.001
NT-proBNP (ng/L)	Before treatment	215.09 ± 36.62	211.53 ± 43.52	0.470	0.639
	After treatment	138.00 ± 24.96^*^	111.53 ± 43.52^*^	3.862	<0.001

Note: * indicates a statistically significant difference from before treatment (*P* < 0.05).

**Table A2:** Cardiac autonomic nervous function before and after treatment in both groups

		Control group (*n* = 50)	Research group (*n* = 69)	*t*	*P*
LE/HF	Before treatment	1.08 ± 0.16	1.04 ± 0.26	0.963	0.338
	After treatment	1.20 ± 0.22^*^	1.45 ± 0.32^*^	4.766	<0.001
SDNN (ms)	Before treatment	47.92 ± 7.51	48.19 ± 9.20	0.170	0.865
	After treatment	50.60 ± 9.08^*^	55.70 ± 8.52^*^	3.135	0.002
RMDSSD (ms)	Before treatment	30.04 ± 6.64	30.51 ± 8.12	0.336	0.738
	After treatment	33.42 ± 7.17^*^	36.46 ± 7.91^*^	2.151	0.034

Note: * indicates a statistically significant difference from before treatment (*P* < 0.05).

**Table A3:** Psychological status before and after treatment in both groups

		Control group (*n* = 50)	Research group (*n* = 69)	*t*	*P*
SAS	Before treatment	31.76 ± 4.48	31.70 ± 4.42	0.073	0.942
	After treatment	28.02 ± 2.66^*^	24.51 ± 3.02^*^	6.574	<0.001
SDS	Before treatment	33.10 ± 3.84	32.72 ± 4.53	0.481	0.632
	After treatment	28.34 ± 2.84^*^	24.80 ± 2.99^*^	6.510	<0.001

Note: * indicates a statistically significant difference from before treatment (*P* < 0.05).

**Table A4:** Self-care ability before and after treatment in both groups

		Control group (*n* = 50)	Research group (*n* = 69)	*t*	*P*
ADL score	Before treatment	53.78 ± 3.71	54.19 ± 2.80	0.687	0.493
	After treatment	62.18 ± 3.93^*^	66.65 ± 3.71^*^	6.328	<0.001
BI	Before treatment	45.78 ± 6.23	45.23 ± 9.06	0.370	0.712
	After treatment	73.52 ± 6.90^*^	80.68 ± 7.43^*^	5.345	<0.001

Note: * indicates a statistically significant difference from before treatment (*P* < 0.05).

**Table A5:** Sleep quality and activity before and after treatment in both groups

		Control group (*n* = 50)	Research group (*n* = 69)	*t*	*P*
PSQI	Before treatment	13.00 ± 3.73	12.74 ± 2.75	0.438	0.662
	After treatment	9.68 ± 1.95^*^	7.42 ± 2.02^*^	6.112	<0.001
6MWT (m)	Before treatment	409.48 ± 43.17	414.03 ± 33.81	0.645	0.521
	After treatment	426.38 ± 27.30^*^	450.68 ± 41.02^*^	3.643	<0.001

Note: * indicates a statistically significant difference from before treatment (*P* < 0.05).
